# Aqua­(4,5-dihydroxy­benzene-1,3-disulfonato-κ*O*)bis­(1,10-phenanthroline-κ^2^ *N,N*’)cadmium(II) monohydrate

**DOI:** 10.1107/S1600536807067918

**Published:** 2008-01-23

**Authors:** Xiangdong Zhang, Chunhua Ge, Lei Guan, Zhimin Sun

**Affiliations:** a College of Chemistry, Liaoning University, Shenyang 110036, People’s Republic of China

## Abstract

In the title compound, [Cd(C_6_H_4_O_8_S_2_)(C_12_H_8_N_2_)_2_(H_2_O)]·H_2_O, each Cd^II^ ion is coordinated by four N atoms [Cd—N = 2.310 (7)–2.341 (7) Å] from two 1,10-phenanthroline ligands, one O atom [Cd—O = 2.300 (6) Å] from a 4,5-dihydroxy­benzene-1,3-disulfonate ligand and one aqua O atom [Cd—O = 2.288 (7) Å] in a distorted octa­hedral geometry. The crystal packing exhibits inter­molecular O—H⋯O and C—H⋯O hydrogen bonds and π–π inter­actions evidenced by relatively short distances [3.525 (5)–3.937 (6) Å] between the centroids of the six-membered rings of neighbouring mol­ecules.

## Related literature

For related literature, see: Haddad & Raymond (1986 ▶); Riley *et al.* (1983 ▶); Sheriff *et al.* (2003 ▶); Sun *et al.* (1995 ▶).
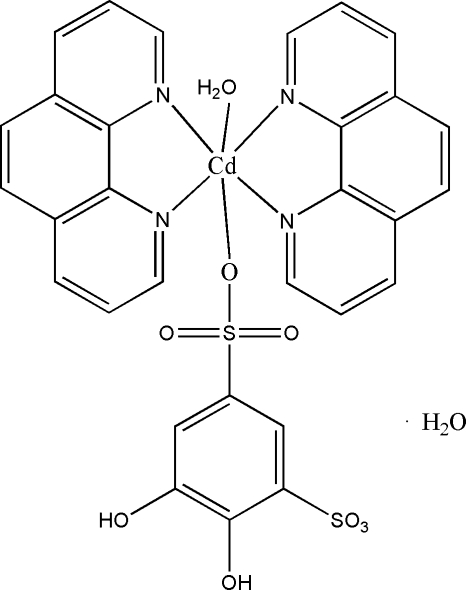


## Experimental

### 

#### Crystal data

[Cd(C_6_H_4_O_8_S_2_)(C_12_H_8_N_2_)_2_(H_2_O)]·H_2_O
*M* *_r_* = 777.05
Monoclinic, 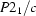

*a* = 16.570 (5) Å
*b* = 9.330 (3) Å
*c* = 24.585 (6) Å
β = 127.199 (16)°
*V* = 3027.5 (15) Å^3^
*Z* = 4
Mo *K*α radiation
μ = 0.93 mm^−1^
*T* = 293 (2) K
0.30 × 0.20 × 0.18 mm

#### Data collection

Bruker SMART CCD area-detector diffractometer
Absorption correction: multi-scan (*SADABS*; Bruker, 2001 ▶) *T*_min_ = 0.816, *T*_max_ = 0.851
6494 measured reflections
5155 independent reflections
3318 reflections with *I* > 2σ(*I*)
*R*_int_ = 0.031

#### Refinement

*R*[*F* ^2^ > 2σ(*F* ^2^)] = 0.067
*wR*(*F* ^2^) = 0.223
*S* = 1.16
5155 reflections
426 parameters
H-atom parameters constrained
Δρ_max_ = 1.32 e Å^−3^
Δρ_min_ = −2.31 e Å^−3^

### 

Data collection: *SMART* (Bruker, 2001 ▶); cell refinement: *SAINT* (Bruker, 2001 ▶); data reduction: *SAINT*; program(s) used to solve structure: *SHELXS97* (Sheldrick, 1997*a* ▶); program(s) used to refine structure: *SHELXL97* (Sheldrick, 1997*a* ▶); molecular graphics: *SHELXTL* (Sheldrick, 1997*b* ▶); software used to prepare material for publication: *SHELXL97*.

## Supplementary Material

Crystal structure: contains datablocks I, global. DOI: 10.1107/S1600536807067918/cv2375sup1.cif

Structure factors: contains datablocks I. DOI: 10.1107/S1600536807067918/cv2375Isup2.hkl

Additional supplementary materials:  crystallographic information; 3D view; checkCIF report

## Figures and Tables

**Table 1:** Centroid-to-centroid distances (Å) *Cg*1, *Cg*2, *Cg*3, *Cg*4, *Cg*5 and *Cg*6 are the centroids of N2/C6/C9–C12, N3/C13–C17, N4/C18/C21–C24, C4–C9, C16–C21 and C25–C30, respectively

*Cg*1⋯*Cg*4^i^	3.734 (7)
*Cg*1⋯*Cg*6^ii^	3.937 (6)
*Cg*2⋯*Cg*6^iii^	3.525 (5)
*Cg*3⋯*Cg*3^iv^	3.900 (7)
*Cg*4⋯*Cg*4^i^	3.533 (7)
*Cg*5⋯*Cg*5^v^	3.606 (7)

Symmetry codes: (i) 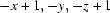; (ii) 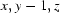; (iii) 
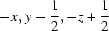
; (iv) 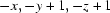; (v) 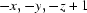.

**Table 2:** Hydrogen-bond geometry (Å, °)

*D*—H⋯*A*	*D*—H	H⋯*A*	*D*⋯*A*	*D*—H⋯*A*
O1—H1*A*⋯O3	0.82	2.15	2.790 (10)	135
O1—H1*B*⋯O5^vi^	0.82	1.93	2.735 (10)	167
O10—H10*A*⋯O6^vii^	0.82	2.22	2.737 (19)	121
O10—H10*B*⋯O7	0.82	2.18	2.876 (15)	143
O8—H8⋯O7	0.82	1.87	2.614 (11)	150
O9—H9⋯O4^viii^	0.82	1.90	2.690 (9)	160
C23—H23⋯O8^ix^	0.93	2.57	3.465 (15)	163
C23—H23⋯O9^ix^	0.93	2.40	3.112 (17)	134

Symmetry codes: (vi) 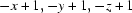; (vii) 
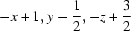
; (viii) 
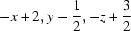
; (ix) 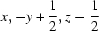.

**Fig. 1.**
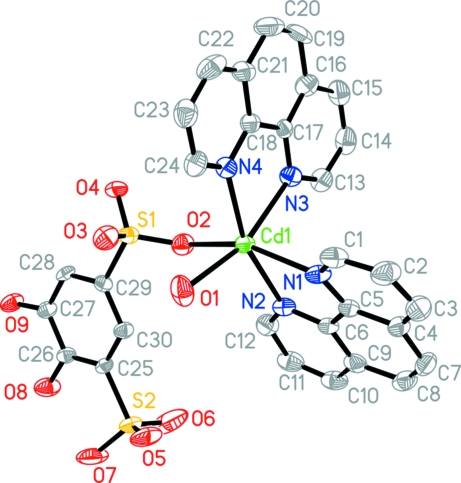
View of (I), showing atomic labels and displacement ellipsoids drawn at the 30% probability level. Uncoordinated water and H atoms are omitted for clarity.

## supplementary crystallographic information

### Comment

As a multi-group compound, 4,5-dihydroxybenzene-1,3-disulfonic acid (H_2_dhds) is a good candidate for investigation of supramolecular assemblies (Haddad & Raymond, 1986; Riley *et al.*, 1983; Sheriff *et al.*, 2003; Sun *et al.*, 1995). Herewith we present the title compound, (I), containing dhds anion as a coordinating ligand.

In (I) (Fig. 1), the dhds anion undoubtedly plays an important role in the formation and stabilization of the three dimesional supramolecular network (Fig.1). Each complex connects with six other complexes by inter-molecular O—H···O and C—H···O hydrogen bonds (Table 2). Significant π···π interactions between pairs of dhds and 1,10-phenanthroline or pairs of 1,10-phenanthrolines exist in (I) (Table 1).

### Experimental

In a typical synthesis, hydrated nitrate (0.5 mmol), phen (1 mmol), 4,5-dihydroxybenzene-1,3-disulfonic acid (0.5 mmol) and NaOH (1 mmol) were mixed in ethanol:H_2_O (v: v = 1:1, 20 ml) solution. The resulting mixture was stirred for 4 h and the solution was filtered. By slow evaporation of the solvent, block-shape single crystals suitable for X-ray analysis were obtained after several weeks.

### Refinement

C-bound H atoms were placed in geometrically idealized positions (C*sp*^2^—H = 0.93 Å) and refined with *U*_iso_(H) =1.2*U*_eq_(C). H atoms attached to O were located from difference Fourier maps, but placed in idealized positions (O—H = 0.82 Å) and refined as riding with *U*_iso_(H) =1.5*U*_eq_(O).

### Crystal data

**Table:**

[Cd(C_6_H_4_O_8_S_2_)(C_12_H_8_N_2_)_2_(H_2_O)]·H_2_O	*F*_000_ = 1568
*M**_r_* = 777.05	*D*_x_ = 1.705 Mg m^−^^3^
Monoclinic, *P*2_1_/*c*	Mo *K*α radiation λ = 0.71073 Å
Hall symbol: -P 2ybc	Cell parameters from 672 reflections
*a* = 16.570 (5) Å	θ = 2.4–22.8º
*b* = 9.330 (3) Å	µ = 0.93 mm^−^^1^
*c* = 24.585 (6) Å	*T* = 293 (2) K
β = 127.199 (16)º	Block, colourless
*V* = 3027.5 (15) Å^3^	0.30 × 0.20 × 0.18 mm
*Z* = 4	

### Data collection

**Table:**

Bruker SMART CCD area-detector diffractometer	5155 independent reflections
Radiation source: fine-focus sealed tube	3318 reflections with *I* > 2σ(*I*)
Monochromator: graphite	*R*_int_ = 0.031
*T* = 293(2) K	θ_max_ = 25.0º
φ and ω scans	θ_min_ = 2.5º
Absorption correction: multi-scan(SADABS; Bruker, 2001)	*h* = −1→19
*T*_min_ = 0.816, *T*_max_ = 0.851	*k* = −11→1
6494 measured reflections	*l* = −29→24

### Refinement

**Table:**

Refinement on *F*^2^	Secondary atom site location: difference Fourier map
Least-squares matrix: full	Hydrogen site location: inferred from neighbouring sites
*R*[*F*^2^ > 2σ(*F*^2^)] = 0.067	H-atom parameters constrained
*wR*(*F*^2^) = 0.223	*w* = 1/[σ^2^(*F*_o_^2^) + (0.084*P*)^2^ + 19.1074*P*] where *P* = (*F*_o_^2^ + 2*F*_c_^2^)/3
*S* = 1.16	(Δ/σ)_max_ < 0.001
5155 reflections	Δρ_max_ = 1.32 e Å^−^^3^
426 parameters	Δρ_min_ = −2.31 e Å^−^^3^
Primary atom site location: structure-invariant direct methods	Extinction correction: none

### Special details

**Table:**

Geometry. All e.s.d.'s (except the e.s.d. in the dihedral angle between two l.s. planes) are estimated using the full covariance matrix. The cell e.s.d.'s are taken into account individually in the estimation of e.s.d.'s in distances, angles and torsion angles; correlations between e.s.d.'s in cell parameters are only used when they are defined by crystal symmetry. An approximate (isotropic) treatment of cell e.s.d.'s is used for estimating e.s.d.'s involving l.s. planes.
Refinement. Refinement of *F*^2^ against ALL reflections. The weighted *R*-factor *wR* and goodness of fit *S* are based on *F*^2^, conventional *R*-factors *R* are based on *F*, with *F* set to zero for negative *F*^2^. The threshold expression of *F*^2^ > σ(*F*^2^) is used only for calculating *R*-factors(gt) *etc*. and is not relevant to the choice of reflections for refinement. *R*-factors based on *F*^2^ are statistically about twice as large as those based on *F*, and *R*- factors based on ALL data will be even larger.

### Fractional atomic coordinates and isotropic or equivalent isotropic displacement parameters (Å^2^)

**Table:**

	*x*	*y*	*z*	*U*_iso_*/*U*_eq_	
Cd1	0.78090 (4)	0.74745 (7)	0.54487 (3)	0.0437 (2)	
S1	0.87188 (16)	0.4361 (3)	0.64067 (11)	0.0466 (5)	
S2	0.59688 (16)	0.4432 (3)	0.69352 (12)	0.0534 (6)	
O1	0.6654 (5)	0.5671 (8)	0.4872 (4)	0.090 (3)	
H1A	0.6817	0.4823	0.4953	0.135*	
H1B	0.6063	0.5669	0.4528	0.135*	
O2	0.8517 (5)	0.5903 (7)	0.6349 (3)	0.0574 (17)	
O3	0.8155 (5)	0.3681 (8)	0.5744 (3)	0.0665 (19)	
O4	0.9804 (5)	0.4051 (8)	0.6832 (4)	0.0633 (19)	
O5	0.5308 (5)	0.4812 (11)	0.6236 (4)	0.084 (3)	
O6	0.6305 (7)	0.5630 (14)	0.7383 (6)	0.149 (6)	
O7	0.5553 (7)	0.3294 (12)	0.7104 (6)	0.123 (4)	
O8	0.7292 (6)	0.1973 (10)	0.7903 (4)	0.072 (2)	
H8	0.6746	0.2320	0.7770	0.108*	
O9	0.8930 (5)	0.0926 (8)	0.8130 (3)	0.0590 (18)	
H9	0.9284	0.0469	0.8062	0.088*	
N1	0.6745 (5)	0.9283 (9)	0.4689 (4)	0.0518 (19)	
N2	0.7183 (5)	0.8708 (9)	0.5940 (4)	0.0498 (18)	
N3	0.9266 (5)	0.8882 (9)	0.5947 (3)	0.0489 (18)	
N4	0.8570 (5)	0.6863 (9)	0.4945 (4)	0.0472 (17)	
C1	0.6530 (7)	0.9512 (13)	0.4087 (5)	0.064 (3)	
H1	0.6733	0.8847	0.3910	0.077*	
C2	0.6005 (8)	1.0726 (15)	0.3710 (6)	0.076 (3)	
H2	0.5879	1.0885	0.3292	0.092*	
C3	0.5669 (8)	1.1702 (14)	0.3957 (6)	0.072 (3)	
H3	0.5320	1.2520	0.3707	0.086*	
C4	0.5858 (7)	1.1449 (11)	0.4582 (5)	0.057 (2)	
C5	0.6410 (6)	1.0214 (10)	0.4939 (5)	0.049 (2)	
C6	0.6605 (6)	0.9861 (10)	0.5582 (5)	0.048 (2)	
C7	0.5465 (8)	1.2313 (11)	0.4849 (7)	0.066 (3)	
H7	0.5101	1.3136	0.4614	0.079*	
C8	0.5600 (7)	1.1980 (13)	0.5419 (6)	0.069 (3)	
H8A	0.5327	1.2569	0.5576	0.082*	
C9	0.6166 (7)	1.0715 (11)	0.5808 (5)	0.054 (2)	
C10	0.6308 (8)	1.0324 (13)	0.6411 (6)	0.067 (3)	
H10	0.6020	1.0862	0.6572	0.080*	
C11	0.6879 (8)	0.9133 (14)	0.6760 (6)	0.066 (3)	
H11	0.6971	0.8839	0.7155	0.080*	
C12	0.7314 (8)	0.8380 (12)	0.6515 (5)	0.062 (3)	
H12	0.7722	0.7598	0.6766	0.074*	
C13	0.9588 (7)	0.9896 (11)	0.6411 (5)	0.057 (2)	
H13	0.9199	1.0091	0.6559	0.069*	
C14	1.0458 (8)	1.0686 (12)	0.6690 (5)	0.071 (3)	
H14	1.0646	1.1392	0.7014	0.086*	
C15	1.1042 (7)	1.0399 (12)	0.6476 (6)	0.071 (3)	
H15	1.1638	1.0905	0.6656	0.085*	
C16	1.0720 (7)	0.9326 (12)	0.5979 (5)	0.062 (3)	
C17	0.9822 (6)	0.8634 (9)	0.5726 (4)	0.0409 (19)	
C18	0.9451 (6)	0.7565 (9)	0.5191 (4)	0.045 (2)	
C19	1.1266 (7)	0.9027 (14)	0.5705 (6)	0.072 (3)	
H19	1.1869	0.9505	0.5878	0.087*	
C20	1.0910 (9)	0.8066 (17)	0.5204 (7)	0.080 (4)	
H20	1.1272	0.7883	0.5035	0.096*	
C21	0.9986 (8)	0.7317 (11)	0.4924 (5)	0.058 (3)	
C22	0.9556 (9)	0.6364 (14)	0.4378 (6)	0.074 (4)	
H22	0.9876	0.6206	0.4177	0.089*	
C23	0.8671 (10)	0.5656 (12)	0.4130 (6)	0.069 (3)	
H23	0.8398	0.4996	0.3776	0.083*	
C24	0.8199 (8)	0.5964 (11)	0.4429 (5)	0.060 (3)	
H24	0.7587	0.5512	0.4257	0.072*	
C25	0.7074 (5)	0.3669 (9)	0.7078 (4)	0.0366 (17)	
C26	0.7595 (6)	0.2558 (9)	0.7554 (4)	0.0394 (18)	
C27	0.8460 (6)	0.1997 (10)	0.7667 (4)	0.044 (2)	
C28	0.8808 (6)	0.2563 (9)	0.7321 (4)	0.0389 (18)	
H28	0.9399	0.2209	0.7405	0.047*	
C29	0.8279 (6)	0.3649 (9)	0.6853 (4)	0.0371 (18)	
C30	0.7408 (6)	0.4223 (10)	0.6727 (4)	0.0425 (19)	
H30	0.7059	0.4963	0.6414	0.051*	
O10	0.3768 (9)	0.3091 (18)	0.7042 (7)	0.171 (6)	
H10A	0.4004	0.2821	0.7429	0.257*	
H10B	0.4159	0.2782	0.6971	0.257*	

### Atomic displacement parameters (Å^2^)

**Table:**

	*U*^11^	*U*^22^	*U*^33^	*U*^12^	*U*^13^	*U*^23^
Cd1	0.0384 (4)	0.0485 (4)	0.0461 (4)	0.0018 (3)	0.0264 (3)	0.0014 (3)
S1	0.0409 (11)	0.0553 (14)	0.0491 (12)	0.0034 (10)	0.0300 (10)	0.0058 (11)
S2	0.0349 (11)	0.0722 (17)	0.0523 (13)	0.0129 (11)	0.0259 (10)	0.0076 (12)
O1	0.054 (4)	0.060 (5)	0.088 (6)	−0.013 (4)	0.007 (4)	0.008 (4)
O2	0.055 (4)	0.063 (4)	0.054 (4)	0.008 (3)	0.033 (3)	0.008 (3)
O3	0.075 (5)	0.076 (5)	0.059 (4)	0.005 (4)	0.046 (4)	−0.004 (4)
O4	0.041 (3)	0.065 (5)	0.092 (5)	0.009 (3)	0.044 (4)	0.013 (4)
O5	0.042 (4)	0.130 (8)	0.071 (5)	0.031 (4)	0.029 (4)	0.031 (5)
O6	0.056 (5)	0.181 (12)	0.154 (9)	0.017 (6)	0.034 (6)	−0.089 (9)
O7	0.078 (6)	0.142 (9)	0.194 (11)	0.045 (6)	0.107 (7)	0.087 (9)
O8	0.062 (4)	0.098 (6)	0.073 (5)	0.017 (4)	0.050 (4)	0.029 (4)
O9	0.054 (4)	0.074 (5)	0.058 (4)	0.027 (3)	0.039 (3)	0.025 (4)
N1	0.034 (4)	0.075 (6)	0.044 (4)	0.002 (4)	0.022 (3)	0.000 (4)
N2	0.041 (4)	0.063 (5)	0.049 (4)	−0.001 (4)	0.029 (3)	−0.004 (4)
N3	0.036 (4)	0.059 (5)	0.039 (4)	−0.002 (3)	0.016 (3)	−0.001 (4)
N4	0.047 (4)	0.049 (4)	0.044 (4)	−0.003 (4)	0.027 (3)	−0.002 (4)
C1	0.051 (5)	0.087 (8)	0.063 (6)	0.002 (6)	0.038 (5)	0.005 (6)
C2	0.058 (6)	0.104 (10)	0.067 (7)	0.019 (7)	0.038 (6)	0.025 (7)
C3	0.052 (6)	0.073 (8)	0.071 (7)	0.008 (6)	0.027 (5)	0.014 (6)
C4	0.043 (5)	0.054 (6)	0.069 (6)	0.002 (4)	0.031 (5)	−0.001 (5)
C5	0.032 (4)	0.050 (6)	0.060 (5)	−0.004 (4)	0.025 (4)	−0.002 (4)
C6	0.032 (4)	0.053 (6)	0.057 (5)	−0.003 (4)	0.026 (4)	−0.001 (4)
C7	0.050 (6)	0.046 (6)	0.084 (8)	0.006 (4)	0.032 (6)	−0.009 (5)
C8	0.044 (5)	0.064 (7)	0.087 (8)	−0.005 (5)	0.034 (6)	−0.026 (6)
C9	0.040 (5)	0.059 (6)	0.057 (5)	−0.017 (4)	0.026 (4)	−0.017 (5)
C10	0.054 (6)	0.084 (8)	0.073 (7)	−0.019 (6)	0.044 (6)	−0.034 (6)
C11	0.067 (6)	0.090 (9)	0.061 (6)	−0.014 (6)	0.048 (6)	−0.017 (6)
C12	0.057 (6)	0.066 (7)	0.058 (6)	0.000 (5)	0.033 (5)	0.004 (5)
C13	0.055 (5)	0.057 (6)	0.046 (5)	−0.004 (5)	0.024 (4)	−0.011 (5)
C14	0.056 (6)	0.052 (6)	0.060 (6)	−0.007 (5)	0.011 (5)	−0.007 (5)
C15	0.039 (5)	0.061 (7)	0.069 (7)	−0.009 (5)	0.010 (5)	0.005 (6)
C16	0.034 (5)	0.070 (7)	0.057 (6)	0.014 (5)	0.016 (4)	0.017 (5)
C17	0.029 (4)	0.034 (4)	0.044 (5)	0.005 (3)	0.015 (4)	0.012 (4)
C18	0.043 (4)	0.049 (5)	0.044 (4)	0.008 (4)	0.027 (4)	0.014 (4)
C19	0.042 (5)	0.087 (9)	0.090 (8)	−0.001 (5)	0.041 (6)	0.039 (7)
C20	0.054 (6)	0.110 (10)	0.092 (9)	0.017 (7)	0.052 (7)	0.023 (8)
C21	0.058 (5)	0.068 (7)	0.061 (6)	0.018 (5)	0.043 (5)	0.032 (5)
C22	0.094 (9)	0.094 (9)	0.064 (7)	0.040 (7)	0.063 (7)	0.024 (7)
C23	0.092 (8)	0.058 (7)	0.065 (7)	0.006 (6)	0.052 (7)	0.006 (5)
C24	0.065 (6)	0.057 (6)	0.054 (6)	0.003 (5)	0.034 (5)	−0.004 (5)
C25	0.028 (4)	0.042 (5)	0.035 (4)	0.005 (3)	0.016 (3)	−0.003 (4)
C26	0.037 (4)	0.051 (5)	0.034 (4)	0.001 (4)	0.023 (3)	−0.007 (4)
C27	0.033 (4)	0.054 (5)	0.037 (4)	0.008 (4)	0.017 (4)	0.004 (4)
C28	0.031 (4)	0.047 (5)	0.040 (4)	0.010 (4)	0.021 (3)	−0.006 (4)
C29	0.032 (4)	0.044 (5)	0.032 (4)	−0.003 (3)	0.018 (3)	0.000 (4)
C30	0.033 (4)	0.051 (5)	0.039 (4)	0.007 (4)	0.020 (4)	0.007 (4)
O10	0.119 (10)	0.232 (16)	0.159 (12)	0.043 (11)	0.082 (9)	0.040 (12)

### Geometric parameters (Å, °)

**Table:**

Cd1—O1	2.285 (7)	C7—H7	0.9300
Cd1—O2	2.299 (6)	C8—C9	1.450 (16)
Cd1—N4	2.310 (7)	C8—H8A	0.9300
Cd1—N2	2.319 (7)	C9—C10	1.400 (15)
Cd1—N1	2.337 (8)	C10—C11	1.372 (16)
Cd1—N3	2.341 (7)	C10—H10	0.9300
S1—O3	1.446 (7)	C11—C12	1.378 (14)
S1—O4	1.461 (6)	C11—H11	0.9300
S1—O2	1.464 (7)	C12—H12	0.9300
S1—C29	1.772 (8)	C13—C14	1.376 (15)
S2—O5	1.415 (7)	C13—H13	0.9300
S2—O6	1.425 (10)	C14—C15	1.380 (17)
S2—O7	1.455 (9)	C14—H14	0.9300
S2—C25	1.791 (7)	C15—C16	1.411 (16)
O1—H1A	0.8205	C15—H15	0.9300
O1—H1B	0.8202	C16—C17	1.377 (13)
O8—C26	1.343 (11)	C16—C19	1.445 (15)
O8—H8	0.8200	C17—C18	1.457 (12)
O9—C27	1.352 (11)	C18—C21	1.406 (13)
O9—H9	0.8200	C19—C20	1.337 (18)
N1—C1	1.312 (12)	C19—H19	0.9300
N1—C5	1.363 (12)	C20—C21	1.425 (16)
N2—C12	1.330 (12)	C20—H20	0.9300
N2—C6	1.353 (12)	C21—C22	1.394 (16)
N3—C13	1.320 (12)	C22—C23	1.370 (17)
N3—C17	1.343 (11)	C22—H22	0.9300
N4—C24	1.322 (12)	C23—C24	1.390 (15)
N4—C18	1.362 (11)	C23—H23	0.9300
C1—C2	1.387 (16)	C24—H24	0.9300
C1—H1	0.9300	C25—C30	1.377 (11)
C2—C3	1.385 (16)	C25—C26	1.403 (11)
C2—H2	0.9300	C26—C27	1.387 (11)
C3—C4	1.390 (15)	C27—C28	1.389 (12)
C3—H3	0.9300	C28—C29	1.378 (11)
C4—C5	1.402 (14)	C28—H28	0.9300
C4—C7	1.422 (15)	C29—C30	1.388 (11)
C5—C6	1.445 (13)	C30—H30	0.9300
C6—C9	1.402 (13)	O10—H10A	0.8197
C7—C8	1.315 (17)	O10—H10B	0.8197
			
Cg1···Cg4^i^	3.734 (7)	Cg3···Cg3^iv^	3.900 (7)
Cg1···Cg6^ii^	3.937 (6)	Cg4···Cg4^i^	3.533 (7)
Cg2···Cg6^iii^	3.525 (5)	Cg5···Cg5^v^	3.606 (7)
			
O1—Cd1—O2	82.9 (3)	C9—C8—H8A	119.3
O1—Cd1—N4	90.6 (3)	C10—C9—C6	118.4 (10)
O2—Cd1—N4	103.4 (3)	C10—C9—C8	122.6 (10)
O1—Cd1—N2	102.6 (3)	C6—C9—C8	118.9 (9)
O2—Cd1—N2	86.8 (3)	C11—C10—C9	119.0 (10)
N4—Cd1—N2	164.4 (3)	C11—C10—H10	120.5
O1—Cd1—N1	95.3 (3)	C9—C10—H10	120.5
O2—Cd1—N1	158.3 (2)	C10—C11—C12	119.0 (10)
N4—Cd1—N1	98.2 (3)	C10—C11—H11	120.5
N2—Cd1—N1	72.5 (3)	C12—C11—H11	120.5
O1—Cd1—N3	161.8 (3)	N2—C12—C11	123.6 (11)
O2—Cd1—N3	95.1 (2)	N2—C12—H12	118.2
N4—Cd1—N3	72.2 (3)	C11—C12—H12	118.2
N2—Cd1—N3	95.4 (3)	N3—C13—C14	124.7 (10)
N1—Cd1—N3	93.1 (3)	N3—C13—H13	117.7
O3—S1—O4	113.4 (4)	C14—C13—H13	117.7
O3—S1—O2	111.6 (4)	C13—C14—C15	118.1 (10)
O4—S1—O2	112.0 (4)	C13—C14—H14	121.0
O3—S1—C29	107.9 (4)	C15—C14—H14	121.0
O4—S1—C29	105.7 (4)	C14—C15—C16	118.8 (10)
O2—S1—C29	105.9 (4)	C14—C15—H15	120.6
O5—S2—O6	113.4 (7)	C16—C15—H15	120.6
O5—S2—O7	112.1 (6)	C17—C16—C15	117.6 (10)
O6—S2—O7	112.6 (8)	C17—C16—C19	120.6 (10)
O5—S2—C25	106.7 (4)	C15—C16—C19	121.6 (11)
O6—S2—C25	106.5 (5)	N3—C17—C16	123.7 (9)
O7—S2—C25	104.9 (5)	N3—C17—C18	117.6 (7)
Cd1—O1—H1A	122.0	C16—C17—C18	118.7 (8)
Cd1—O1—H1B	132.2	N4—C18—C21	121.5 (9)
H1A—O1—H1B	105.1	N4—C18—C17	119.0 (7)
S1—O2—Cd1	132.3 (4)	C21—C18—C17	119.5 (8)
C26—O8—H8	109.5	C20—C19—C16	120.4 (10)
C27—O9—H9	109.5	C20—C19—H19	119.8
C1—N1—C5	119.9 (9)	C16—C19—H19	119.8
C1—N1—Cd1	125.3 (7)	C19—C20—C21	121.5 (11)
C5—N1—Cd1	114.5 (6)	C19—C20—H20	119.3
C12—N2—C6	118.2 (8)	C21—C20—H20	119.3
C12—N2—Cd1	127.0 (7)	C22—C21—C18	117.1 (10)
C6—N2—Cd1	114.8 (6)	C22—C21—C20	123.6 (10)
C13—N3—C17	117.1 (8)	C18—C21—C20	119.3 (11)
C13—N3—Cd1	127.2 (7)	C23—C22—C21	121.5 (10)
C17—N3—Cd1	115.7 (6)	C23—C22—H22	119.3
C24—N4—C18	118.9 (8)	C21—C22—H22	119.3
C24—N4—Cd1	125.6 (7)	C22—C23—C24	117.3 (11)
C18—N4—Cd1	115.4 (6)	C22—C23—H23	121.4
N1—C1—C2	121.3 (11)	C24—C23—H23	121.4
N1—C1—H1	119.4	N4—C24—C23	123.7 (10)
C2—C1—H1	119.4	N4—C24—H24	118.2
C3—C2—C1	120.0 (11)	C23—C24—H24	118.2
C3—C2—H2	120.0	C30—C25—C26	121.5 (7)
C1—C2—H2	120.0	C30—C25—S2	118.6 (6)
C2—C3—C4	119.5 (11)	C26—C25—S2	119.8 (6)
C2—C3—H3	120.3	O8—C26—C27	117.3 (8)
C4—C3—H3	120.3	O8—C26—C25	123.7 (7)
C3—C4—C5	117.2 (10)	C27—C26—C25	119.0 (7)
C3—C4—C7	123.7 (10)	O9—C27—C26	116.5 (8)
C5—C4—C7	119.0 (10)	O9—C27—C28	123.7 (7)
N1—C5—C4	122.0 (9)	C26—C27—C28	119.7 (8)
N1—C5—C6	117.8 (8)	C29—C28—C27	120.1 (7)
C4—C5—C6	120.1 (9)	C29—C28—H28	120.0
N2—C6—C9	121.8 (9)	C27—C28—H28	120.0
N2—C6—C5	119.7 (8)	C28—C29—C30	121.3 (7)
C9—C6—C5	118.5 (9)	C28—C29—S1	120.1 (6)
C8—C7—C4	121.8 (11)	C30—C29—S1	118.6 (6)
C8—C7—H7	119.1	C25—C30—C29	118.3 (8)
C4—C7—H7	119.1	C25—C30—H30	120.9
C7—C8—C9	121.4 (10)	C29—C30—H30	120.9
C7—C8—H8A	119.3	H10A—O10—H10B	104.8
			
O3—S1—O2—Cd1	−16.4 (7)	N2—C6—C9—C8	176.7 (8)
O4—S1—O2—Cd1	111.8 (5)	C5—C6—C9—C8	−4.7 (12)
C29—S1—O2—Cd1	−133.5 (5)	C7—C8—C9—C10	−179.0 (10)
O1—Cd1—O2—S1	44.5 (6)	C7—C8—C9—C6	2.2 (15)
N4—Cd1—O2—S1	−44.4 (6)	C6—C9—C10—C11	0.7 (14)
N2—Cd1—O2—S1	147.6 (5)	C8—C9—C10—C11	−178.1 (9)
N1—Cd1—O2—S1	130.8 (7)	C9—C10—C11—C12	1.5 (15)
N3—Cd1—O2—S1	−117.3 (5)	C6—N2—C12—C11	1.2 (15)
O1—Cd1—N1—C1	−76.7 (8)	Cd1—N2—C12—C11	−177.0 (8)
O2—Cd1—N1—C1	−160.6 (8)	C10—C11—C12—N2	−2.6 (16)
N4—Cd1—N1—C1	14.7 (8)	C17—N3—C13—C14	−1.5 (15)
N2—Cd1—N1—C1	−178.2 (9)	Cd1—N3—C13—C14	179.5 (8)
N3—Cd1—N1—C1	87.1 (8)	N3—C13—C14—C15	−0.3 (17)
O1—Cd1—N1—C5	108.9 (6)	C13—C14—C15—C16	0.6 (16)
O2—Cd1—N1—C5	25.0 (11)	C14—C15—C16—C17	1.0 (15)
N4—Cd1—N1—C5	−159.7 (6)	C14—C15—C16—C19	176.5 (10)
N2—Cd1—N1—C5	7.4 (6)	C13—N3—C17—C16	3.3 (13)
N3—Cd1—N1—C5	−87.3 (6)	Cd1—N3—C17—C16	−177.7 (7)
O1—Cd1—N2—C12	82.3 (8)	C13—N3—C17—C18	−177.2 (8)
O2—Cd1—N2—C12	0.3 (8)	Cd1—N3—C17—C18	1.9 (9)
N4—Cd1—N2—C12	−131.1 (10)	C15—C16—C17—N3	−3.0 (14)
N1—Cd1—N2—C12	173.9 (8)	C19—C16—C17—N3	−178.6 (8)
N3—Cd1—N2—C12	−94.5 (8)	C15—C16—C17—C18	177.4 (8)
O1—Cd1—N2—C6	−96.0 (6)	C19—C16—C17—C18	1.8 (13)
O2—Cd1—N2—C6	−178.0 (6)	C24—N4—C18—C21	−2.1 (13)
N4—Cd1—N2—C6	50.6 (12)	Cd1—N4—C18—C21	−178.9 (6)
N1—Cd1—N2—C6	−4.4 (6)	C24—N4—C18—C17	175.5 (8)
N3—Cd1—N2—C6	87.2 (6)	Cd1—N4—C18—C17	−1.2 (10)
O1—Cd1—N3—C13	−163.2 (9)	N3—C17—C18—N4	−0.5 (11)
O2—Cd1—N3—C13	−80.4 (8)	C16—C17—C18—N4	179.1 (8)
N4—Cd1—N3—C13	177.1 (8)	N3—C17—C18—C21	177.2 (8)
N2—Cd1—N3—C13	6.8 (8)	C16—C17—C18—C21	−3.2 (12)
N1—Cd1—N3—C13	79.5 (8)	C17—C16—C19—C20	−0.4 (16)
O1—Cd1—N3—C17	17.8 (12)	C15—C16—C19—C20	−175.8 (11)
O2—Cd1—N3—C17	100.6 (6)	C16—C19—C20—C21	0.3 (18)
N4—Cd1—N3—C17	−1.8 (6)	N4—C18—C21—C22	2.5 (13)
N2—Cd1—N3—C17	−172.1 (6)	C17—C18—C21—C22	−175.2 (8)
N1—Cd1—N3—C17	−99.4 (6)	N4—C18—C21—C20	−179.3 (9)
O1—Cd1—N4—C24	11.1 (8)	C17—C18—C21—C20	3.1 (13)
O2—Cd1—N4—C24	93.9 (8)	C19—C20—C21—C22	176.4 (11)
N2—Cd1—N4—C24	−136.4 (10)	C19—C20—C21—C18	−1.7 (17)
N1—Cd1—N4—C24	−84.3 (8)	C18—C21—C22—C23	−2.6 (15)
N3—Cd1—N4—C24	−174.9 (8)	C20—C21—C22—C23	179.3 (11)
O1—Cd1—N4—C18	−172.4 (6)	C21—C22—C23—C24	2.3 (16)
O2—Cd1—N4—C18	−89.6 (6)	C18—N4—C24—C23	1.8 (15)
N2—Cd1—N4—C18	40.1 (12)	Cd1—N4—C24—C23	178.2 (8)
N1—Cd1—N4—C18	92.2 (6)	C22—C23—C24—N4	−1.9 (16)
N3—Cd1—N4—C18	1.6 (6)	O5—S2—C25—C30	35.7 (8)
C5—N1—C1—C2	3.1 (15)	O6—S2—C25—C30	−85.7 (9)
Cd1—N1—C1—C2	−171.0 (8)	O7—S2—C25—C30	154.8 (8)
N1—C1—C2—C3	−2.1 (17)	O5—S2—C25—C26	−146.1 (7)
C1—C2—C3—C4	−0.3 (17)	O6—S2—C25—C26	92.5 (9)
C2—C3—C4—C5	1.5 (15)	O7—S2—C25—C26	−27.0 (9)
C2—C3—C4—C7	−173.9 (10)	C30—C25—C26—O8	−179.2 (8)
C1—N1—C5—C4	−1.8 (13)	S2—C25—C26—O8	2.6 (12)
Cd1—N1—C5—C4	172.9 (7)	C30—C25—C26—C27	−0.7 (12)
C1—N1—C5—C6	175.7 (8)	S2—C25—C26—C27	−178.9 (6)
Cd1—N1—C5—C6	−9.6 (10)	O8—C26—C27—O9	−1.2 (12)
C3—C4—C5—N1	−0.5 (14)	C25—C26—C27—O9	−179.8 (8)
C7—C4—C5—N1	175.2 (9)	O8—C26—C27—C28	−179.9 (8)
C3—C4—C5—C6	−178.0 (9)	C25—C26—C27—C28	1.5 (13)
C7—C4—C5—C6	−2.3 (13)	O9—C27—C28—C29	179.5 (8)
C12—N2—C6—C9	1.2 (13)	C26—C27—C28—C29	−1.9 (13)
Cd1—N2—C6—C9	179.7 (6)	C27—C28—C29—C30	1.5 (12)
C12—N2—C6—C5	−177.3 (8)	C27—C28—C29—S1	−179.5 (6)
Cd1—N2—C6—C5	1.1 (10)	O3—S1—C29—C28	98.1 (7)
N1—C5—C6—N2	5.9 (12)	O4—S1—C29—C28	−23.4 (8)
C4—C5—C6—N2	−176.6 (8)	O2—S1—C29—C28	−142.3 (7)
N1—C5—C6—C9	−172.7 (8)	O3—S1—C29—C30	−82.8 (8)
C4—C5—C6—C9	4.9 (13)	O4—S1—C29—C30	155.6 (7)
C3—C4—C7—C8	175.0 (11)	O2—S1—C29—C30	36.7 (8)
C5—C4—C7—C8	−0.4 (15)	C26—C25—C30—C29	0.3 (12)
C4—C7—C8—C9	0.4 (16)	S2—C25—C30—C29	178.5 (6)
N2—C6—C9—C10	−2.2 (13)	C28—C29—C30—C25	−0.7 (12)
C5—C6—C9—C10	176.4 (8)	S1—C29—C30—C25	−179.7 (6)

Symmetry codes: (i) −*x*+1, −*y*, −*z*+1; (ii) *x*, *y*−1, *z*; (iii) −*x*, *y*−1/2, −*z*+1/2; (iv) −*x*, −*y*+1, −*z*+1; (v) −*x*, −*y*, −*z*+1.

### Hydrogen-bond geometry (Å, °)

**Table:**

*D*—H···*A*	*D*—H	H···*A*	*D*···*A*	*D*—H···*A*
O1—H1A···O3	0.82	2.15	2.790 (10)	135
O1—H1B···O5^vi^	0.82	1.93	2.735 (10)	167
O10—H10A···O6^vii^	0.82	2.22	2.737 (19)	121
O10—H10B···O7	0.82	2.18	2.876 (15)	143
O8—H8···O7	0.82	1.87	2.614 (11)	150
O9—H9···O4^viii^	0.82	1.90	2.690 (9)	160
C23—H23···O8^ix^	0.93	2.57	3.465 (15)	163
C23—H23···O9^ix^	0.93	2.40	3.112 (17)	134

Symmetry codes: (vi) −*x*+1, −*y*+1, −*z*+1; (vii) −*x*+1, *y*−1/2, −*z*+3/2; (viii) −*x*+2, *y*−1/2, −*z*+3/2; (ix) *x*, −*y*+1/2, *z*−1/2.
